# Sociodemographic factors are associated with dietary patterns in Mexican schoolchildren

**DOI:** 10.1017/S1368980017003299

**Published:** 2017-12-01

**Authors:** Claudia Gabriela García-Chávez, Sonia Rodríguez-Ramírez, Juan A Rivera, Eric Monterrubio-Flores, Katherine L Tucker

**Affiliations:** 1 Center for Research in Nutrition and Health, National Institute of Public Health, Av. Universidad 655, Col. Sta. María Ahuacatitlán, Oficce 115, Cuernavaca, Morelos, México, CP 62100; 2 Department of Clinical Laboratory and Nutritional Sciences, University of Massachusetts, Lowell, MA, USA

**Keywords:** Dietary patterns, Schoolchildren, National survey, Sociodemographic factors

## Abstract

**Objective:**

Childhood obesity has increased rapidly in Mexico, along with changes in the food environment. However, little is known about the dietary patterns (DP) of Mexican children. We aimed to characterize the DP of schoolchildren and to analyse their associations with sociodemographic factors.

**Design:**

Cross-sectional analysis. Dietary and sociodemographic information was obtained, including a single 24 h recall, socio-economic status (SES), geographic region, area of residence and ethnicity. DP were defined with cluster analysis (using *k*-means). Multinomial logistic regression models, adjusted for the survey design, were used to assess associations between DP and sociodemographic variables.

**Setting:**

2012 Mexican National Health and Nutrition Survey (ENSANUT-2012).

**Subjects:**

Schoolchildren (*n* 2751) aged 5–11 years who participated in ENSANUT-2012.

**Results:**

Four DP were identified: ‘Traditional’, ‘Industrialized’, ‘Varied’ and ‘Modern’. Reported energy intake (mean (sd)) was lowest in the ‘Traditional’ and highest in the ‘Industrialized’ DP (7037 (3707) kJ/d (1682 (886) kcal/d) *v*. 8427 (3753) kJ/d (2014 (897) kcal/d), respectively, *P*<0·05). Significant differences in fat and fibre intakes were seen across DP. Non-indigenous *v*. indigenous children were 22·0 times (95 % CI 5·1, 93·6) more likely to have a ‘Modern’ rather than ‘Traditional’ DP. Relative likelihood of having an ‘Industrialized’ rather than ‘Traditional’ DP was 6·2 (95 % CI 3·9, 9·9) among schoolchildren from high SES *v*. low SES.

**Conclusions:**

Among Mexican schoolchildren, DP were associated with sociodemographic variables. Non-indigenous children were significantly more likely to consume a ‘Modern’ than a ‘Traditional’ DP. Children with higher SES were more likely to have an ‘Industrialized’ pattern. It is necessary to consider dietary characteristics in the different sociodemographic strata when dietary interventions are designed.

Mexico and other Latin American countries are experiencing a nutrition transition, characterized by rapidly increasing urbanization and economic growth^(^ ^1^ ^)^, that has led to changes in dietary patterns (DP), eating habits and lifestyles^(^ ^2^ ^)^. Until recently, the Mexican diet was based primarily on locally produced vegetable, tuber and animal foods; but recently food preferences have shifted towards packaged and processed foods (defined as products made by adding sugar, oil and salt, whose main purpose is to increase their durability or enhance their sensory qualities)^(^ ^3^ ^)^, including ready-to-eat or ready-to-heat foods^(^ ^4^ ^)^.

Given the complexity of dietary intake, particularly with respect to rapid changes in food consumption, nutrition epidemiology has moved towards the study of DP as a way to evaluate the cumulative and complex effects of food and nutrient exposures on health^(^ ^5^ ^)^. Characterization of DP may vary across populations due to differences in food availability, residence area, geographic region, cultural practices, ethnically based practices and socio-economic status (SES)^(^ ^6^ ^,^ ^7^ ^)^.

During childhood, poverty is one of the main causes of poor diet quality in some countries^(^ ^2^ ^)^. In addition to SES, family environment can have an impact on children’s food intake^(^ ^8^ ^)^. Parents influence the eating habits of their children, as they are responsible for food availability in the home, and influence the food consumption preferences of the child^(^ ^8^ ^,^ ^9^ ^)^. There is evidence that parental education is related to the selection and intake of healthy *v*. unhealthy foods by their children^(^ ^10^ ^,^ ^11^ ^)^. In Sweden, children of parents with low education had higher intakes of fruits, lower intakes of vegetables, and higher intakes of many unhealthy foods, compared with children of parents with higher education^(^ ^10^ ^)^.

Examining dietary intake with DP allows characterization of individual diets and evaluation of associations between diet and disease^(^ ^12^ ^)^. This is particularly important, as more Mexican children and adolescents are becoming overweight or obese. The results from national surveys show that the prevalence of overweight and obesity in Mexican schoolchildren increased from 18·6 % in 1999 to 34·4 % in 2012^(^ ^13^ ^,^ ^14^ ^)^. The most recent data, from a national survey in 2016, reported a stabilization in this combined national prevalence at 33·4 %, which did not differ statistically from the 34·4 % in 2012^(^ ^15^ ^)^.

Consumption of foods with high energy density and low nutrient content has been related to the onset of non-communicable diseases later in life^(^ ^16^ ^)^. It is important to identify DP in children as a first step in the establishment of interventions to promote needed dietary changes. Therefore, the present study aimed to identify the DP of Mexican schoolchildren and to assess their associations with sociodemographic factors.

## Methods

### Design and study population

The present study is a cross-sectional analysis of data of children aged 5–11 years from the 2012 Mexican National Health and Nutrition Survey (ENSANUT-2012). ENSANUT is a probabilistic survey with national, regional and urban/rural strata designed to be nationally representative. Dietary data are available for urban and rural strata and three regions^(^ ^14^ ^)^.

### Dietary assessment

Dietary data were obtained by 24 h recall. Details on all foods and beverages consumed by the individual the day before the interview were obtained using a multistep method, as follows: (i) obtain a preliminary list of foods consumed throughout the day; (ii) review the food list with queries for foods often overlooked; (iii) complete details for the list of foods, including meal times and associated activities; (iv) complete details for each food, including portion size and recipes; and (v) final review. The details of this method have been published previously^(^ ^17^ ^)^.

The mother, caregiver or person in charge of feeding the child completed the recall. All foods and beverages were queried, including those consumed outside the home.

### Sociodemographic factors

Information on sociodemographic characteristics, age (years), gender of the child and of the head of household, region of the country, urban *v*. rural area of residence, indigenous status, education level of the mother and household head, and SES were obtained through questionnaire.

The country of Mexico was divided into three major regions: (i) North, comprising Baja California, Baja California Sur, Coahuila, Chihuahua, Durango, Nuevo León, Sonora and Tamaulipas; (ii) Centre, comprising Aguascalientes, Colima, Guanajuato, Jalisco, Ciudad de México, Michoacán, Morelos, Nayarit, Querétaro, San Luis Potosí, Sinaloa, Zacatecas and Distrito Federal; and (iii) South, comprising Campeche, Chiapas, Guerrero, Hidalgo, Oaxaca, Puebla, Quintana Roo, Tabasco, Tlaxcala, Veracruz and Yucatán^(^ ^14^ ^)^.

Area of residence was identified as rural for towns with fewer than 2500 inhabitants and urban for towns with 2500 or more inhabitants. Children were classified as indigenous when either parent spoke an indigenous language. The education level of the child’s mother and head of household were defined by the degree achieved or grade completed. An index for SES was constructed using principal components analysis with eight variables: building materials for the floor, walls and ceiling, number of rooms for sleeping, water source, car ownership, number of household goods (refrigerator, washing machine, microwave, stove and boiler) and number of electrical appliances (television, cable television, radio, telephone and computer) owned. The first component explained 40·5 % of the total variability, with an eigenvalue (*λ*) of 3·24, and was selected as the index (I Méndez, National Institute of Public Health, Cuernavaca, Mexico, personal communication, 2014). For the current analysis, the index was divided into tertiles (low, middle and high SES)^(^ ^18^ ^)^.

Food intake was estimated in grams. Daily energy and nutrient intakes were calculated from these, based on the Mexican National Institute of Public Health’s food composition tables (Nutrient Data Base 2012; compilation of the National Institute of Public Health, unpublished results). Also, we estimated fruit and vegetables consumption as the sum of each food include into the food group of fruits and vegetables, by DP.

The total sample includes 2783 children. A total of thirty children were excluded from the analysis after the cleaning process based on energy intake (below –3 sd of the log ratio of energy intake to estimated energy requirement, *n* 29; and above +3 sd, *n* 1), and two more children were also excluded for reporting the consumption of two foods or fewer. Details of the cleaning process are available elsewhere^(^ ^17^ ^)^.

The analytic sample therefore consisted of 2751 children between 5 and 11 years of age, with dietary information.

### Dietary patterns

Thirty-three food groups were created based on nutrient similarity (see online supplementary material, Supplemental Table 1). Some foods groups were similar to those performed by Deming *et al*.^(^ ^19^ ^)^. The percentage of energy consumed from each food group was estimated. These data were standardized by subtracting the mean and dividing by the sd, for use in cluster analysis, using *k*-means convergence, to classify children into non-overlapping groups based on eating patterns^(^ ^20^ ^)^.

Configurations of two- to five-cluster solutions were tested. The solution that best discriminated across groups, while maintaining a sufficient number of cases in each group, was selected. To define DP, foods contributing >2 % of energy and with a percentage energy contribution that differed significantly from other groups were identified. After this identification, each cluster was characterized by describing the average consumption of energy, macronutrients (carbohydrates, protein and fat) and fibre. Patterns were named based on the major food groups and nutrients that characterized the differences in each group relative to others.

### Statistical analysis

Descriptive statistics were obtained. Continuous variables are presented as means with standard deviations, and categorical variables as percentages with confidence intervals. Comparisons between proportions were performed by difference of proportions for independent populations. Robust regression modelling was used to test differences in nutrient intakes across DP^(^ ^21^ ^)^. Multinomial logistic regression was used to assess associations between sociodemographic variables and DP. For this analysis, we assigned the pattern with the features of a traditional Mexican diet (‘Traditional’) as the reference; and selected sociodemographic variables that characterized this pattern (South region, rural area, indigenous population and low SES) as reference categories. The final model included the sociodemographic variables, with the exclusion of the education levels of the mother and the head of household, due to high correlation with SES (variance inflation factor ≥5). All analyses were completed using the statistical software package Stata version 12.0 and were adjusted for the complex sampling design of the survey, considering the definition of primary units, strata and the weighting of the sample^(^ ^21^ ^,^ ^22^ ^)^. The 95 % CI were estimated, and differences were considered statistically significant at *P*<0·05. The Bonferroni method was used to adjust for multiple comparisons, multiplying the observed *P* value by the total number of comparisons^(^ ^23^ ^)^.

### Ethical considerations

Parents or guardians of the children signed informed consent forms before the implementation of the survey and the children were asked for their assent to participate. The Ethics Committee of the National Institute of Public Health approved the ENSANUT-2012 and the consent form.

## Results

The children’s mean age was 8·7 (sd 2·0) years (data not shown). Approximately half were boys and half girls (Table 1). About a third (29·0 %) lived in rural areas and 47·4 % lived in the Centre region, followed by the South region, with 34·8 %.

**Table 1:** Sociodemographic characteristics, overall and by dietary pattern, of school-aged children (5–11 years) from the 2012 Mexican National Health and Nutrition Survey (*n* 2751)

	Sample	Traditional^a^ (*n* 752; 24·4 %)		Industrialized^b^ (*n* 541; 19·4 %)		Varied^c^ (*n* 1154; 43·9 %)		Modern^d^ (*n* 304; 12·2 %)	
	%	%	95 % CI	%	95 % CI	%	95 % CI	%	95 % CI
Sex									
Boys	50·5	25·7^c,d^	22·7, 28·9	19·7^c,d^	16·7, 23·1	42·7^a,b,d^	38·7, 46·8	11·8ª^,b,c^	9·6, 14·6
Girl	49·5	23·1^c,d^	20·3, 26·2	19·1^c,d^	16·5, 22·0	45·1^a,b,d^	41·3, 49·0	12·5^a,b,c^	10·1, 15·5
Region									
South	34·8	33·5^b,c,d^	29·6, 37·3	14·7^a,c^	11·6, 17·6	41·1^a,b,c^	37·2, 45·1	10·7^a,c^	8·4, 13·0
Centre	47·4	18·4^c^	15·2, 21·5	20·2^c^	16·8, 23·5	47·2^a,b,d^	42·3, 52·0	14·3^c^	11·0, 17·4
North	17·8	22·8^c,d^	18·5, 27·1	26·7^c,d^	22·3, 31·1	40·8^a,b,d^	35·6, 45·9	9·6^a,b,c^	6·8, 12·4
Area†									
Rural	29·0	42·2^b,d^	37·8, 46·5	11·3^a,c^	8·9, 13·6	39·0^b,d^	35·1, 43·0	7·5^a,c^	5·5, 9·5
Urban	71·0	17·2^b,c^	14·8, 19·6	22·7^a,c,d^	20·0, 25·4	46·0^a,b,d^	42·3, 49·5	14·1^b,c^	11·7, 16·5
Indigenous population									
Yes	5·6	69·2^b,c,d^	60·8, 77·6	6·7^a,c^	2·3, 11·0	23·3^a,b,d^	15·9, 30·7	0·7^a,c^	0·2, 1·7
No	94·4	21·8^c,d^	19·6, 23·9	20·2^c,d^	18·0, 22·3	45·2^a,b,d^	42·2, 48·1	12·9^a,b,c^	11·0, 14·7
SES‡									
Low	34·9	43·2^b,d^	38·8, 47·4	11·0^a,c^	8·4, 13·6	36·0^b,d^	31·5, 40·2	9·8^a,c^	6·8, 12·8
Middle	34·9	18·1^c^	14·7, 21·6	22·0^c,d^	18·0, 25·8	47·3^a,b,d^	42·3, 52·2	12·6^b,c^	9·7, 15·3
High	30·1	10·0^b,c^	7·4, 12·4	26·2^a,c,d^	21·9, 30·4	49·3^a,b,d^	44·1, 54·5	14·5^b,c^	10·7, 18·2
Mother’s educational level§									
None║	5·2	47·4^b,d^	35·1, 59·6	5·2^a,c^	0·8, 9·64	39·3^b,d^	26·4, 52·0	8·1^a,c^	2·3, 13·9
Elementary school	34·6	35·0^b,d^	30·6, 39·3	14·6^a,c,d^	11·4, 17·7	41·5^b,d^	36·6, 46·3	8·8^a,b,c^	6·3, 11·3
Middle school	37·3	20·6^c^	16·9, 24·2	19·8^c^	16·2, 23·3	43·9^a,b,d^	38·9, 48·9	15·7^c^	11·9, 19·3
High school¶	18·1	8·9^b,c^	5·8, 11·9	28·3^a,c,d^	22·3, 34·3	49·2^a,b,d^	42·8, 55·4	13·6^b,c^	9·5, 17·6
Bachelor’s or higher	4·9	6·2^b,c^	1·9, 10·4	34·3^a,d^	23·9, 44·5	49·6^a,d^	38·3, 60·9	9·9^b,c^	3·4, 16·2

SES, socio-economic status. ^a,b,c,d^Percentages were significantly different between the dietary patterns (*P*<0·05, adjusted by Bonferroni method).

† Rural, <2500 inhabitants; urban, ≥2500 inhabitants.

‡ Calculated with principal components analysis; includes household characteristics, goods and services.

§ *n* 150 observations with data missing.

║ Includes pre-school level.

¶ Includes technical or commercial studies.

There were no significant differences in sex or age within DP (data not shown). Fewer than 6 % of children were in households with indigenous status. Approximately 37 % of mothers had middle school education, followed by elementary school education (34·6 %).

We identified four DP: ‘Traditional’, ‘Industrialized’, ‘Varied’ and ‘Modern’. Table 2 presents the energy contribution (expressed as percentage of energy) of food groups to the diet and indicates differences between DP in the energy contribution. The food groups characterizing the ‘Traditional’ pattern (percentage of energy higher than in the other DP) were: tortilla (29·2 %), legumes (10·9 %), egg (8·0 %), sugar-sweetened beverages (7·6 %), and bread and other cereals (5·1 %). Foods characterizing the ‘Industrialized’ pattern were: milk drinks with sugar (11·2 %), snacks made from flour, corn or potato (11·0 %), fast food (9·8 %), desserts, pastries and sweets (8·9 %), and industrialized beverages (4·6 %). The ‘Varied’ pattern included mainly: meals made of tortilla or corn dough (19·4 %), cereals with sugar (including Mexican sweet bread; 15·2 %), meat and sausage (9·4 %), dairy drinks (5·1 %), fruits (3·5 %), and rice and pasta (2·7 %). The ‘Modern’ pattern included tortas and sandwiches (26·8 %) and breakfast cereals with sugar (4·3 %).

**Table 2:** Contribution (%) of food groups to daily energy intake, by dietary pattern, among school-aged children (5–11 years) from the 2012 Mexican National Health and Nutrition Survey (*n* 2751)

	Traditional^a^ (*n* 752)		Industrialized^b^ (*n* 541)		Varied^c^ (*n* 1154)		Modern^d^ (*n* 304)	
	Mean	sd	Mean	sd	Mean	sd	Mean	sd
Tortilla	29·2^b,c,d^	20·7	6·5^a^	7·8	7·3^a^	8·3	8·0^a^	8·4
Legumes	10·9^b,c,d^	15·6	1·7^a^	4·4	2·1^a^	4·5	1·7^a^	4·6
Egg	8·0^b,c,d^	11·2	3·3^a^	5·5	2·3^a^	4·8	2·2^a^	4·4
Sugar-sweetened beverages	7·6^b,d^	11·9	3·0^a,c^	5·1	5·8^b,d^	9·1	3·9^a,c^	6·5
Bread and other cereals†	5·1^b,c^	12·6	2·8^a^	6·8	1·9^a^	5·1	3·6	7·4
Milk drinks with sugar	1·3^b,c,d^	4·9	11·2^a,c,d^	13·9	2·4^a,b^	5·5	4·4^a,b^	7·8
Snacks made from flour‡, corn or potato	2·1^b^	6·2	11·0^a,c,d^	15·6	2·5^b^	5·3	2·7^b^	5·8
Fast food	0·2^b^	1·8	9·8^a,c,d^	17·2	0·5^b^	3·1	0·3^b^	2·8
Desserts, pastries and sweets	1·7^b^	4·6	8·9^a,c,d^	14·0	1·9^b^	3·8	2·1^b^	4·6
Industrialized beverages	2·9^b^	5·2	4·6^a,c^	6·6	3·4^b^	5·2	3·8	5·2
Meals made of tortilla or corn dough	3·1^b,c^	9·2	9·2^a,c^	13·8	19·4^a,b,d^	22·9	6·0^c^	12·3
Cereals with sugar	6·3^c^	10·3	4·5^c,d^	7·5	15·2^a,b,d^	15·9	7·3^b,c^	9·8
Meat and sausages	5·0^b,c^	11·0	7·8^a^	11·4	9·4^a,d^	14·7	5·3^c^	8·9
Dairy drinks	1·9^c^	4·8	1·5^c^	3·7	5·1^a,b^	7·9	3·4	6·6
Fruits	2·7	5·8	2·2	5·2	3·5^d^	6·8	2·2^c^	4·6
Rice and pasta	1·7^b^	5·3	1·0^a,c^	3·1	2·7^b^	7·1	2·5	5·4
Tortas§ and sandwich	0·5^b,d^	2·5	1·6^a,d^	4·5	0·9^d^	3·2	26·8^a,b,c^	12·3
Breakfast cereal with sugar	0·6^b,c,d^	3·3	1·5^a,c,d^	4·9	3·5^a,b^	9·5	4·3^a,b^	10·2
Vegetable-based stews	0·8^b^	3·9	0·2^a,d^	1·0	0·3	2·2	1·2^b^	4·0
Vegetables	0·2^b,c^	1·1	0·6^a^	3·1	0·4^a^	1·6	0·3	1·6
Fish and seafood	0·5	3·9	0·7	3·8	1·6	7·6	0·4	2·4
Yoghurt	0·3^b,c^	2·2	1·2^a^	4·1	0·9^a^	3·2	1·1	3·5
Drinkable yoghurt	0·2	1·8	0·4	2·4	0·5	2·5	1·9	5·5
Cheeses	0·9^b,c,d^	4·1	0·1^a^	0·6	0·1^a^	0·7	0·1^a^	0·5
Juices	1·0	0·8	0·2	1·2	0·2	1·8	0·2	1·1
Soups and broths	4·0	8·8	2·6	6·4	3·9	8·7	2·8	5·9
Potato	0·8	4·2	1·0	3·8	1·7^d^	7·7	0·4^c^	2·7
Seeds and oils	0·4	3·1	0·5	3·3	0·2	1·6	0·8	4·2
Miscellaneous	0·6^b,c,d^	3·6	0·1^a^	0·8	0·1^a^	0·9	0·1^a^	0·5

The food groups that did not contribute energy, and hence were not included in the analysis, were drinking-water, unsweetened drinks, diet sodas, and supplements and dietary supplements. ^a,b,c,d^Mean values were significantly different between the dietary patterns, adjusted for the survey design (*P*<0·05, adjusted by Bonferroni method).

† Includes salty bread, wholemeal bread. Excludes corn, rice, pasta and cereals with sugar.

‡ Wheat flour.

§ Mexican typical food, similar to a sandwich, made with crusty bread and served cold or hot.

The lowest energy intake was seen in the ‘Traditional’ pattern (7037 (sd 3707) kJ/d (1682 (sd 886) kcal/d)) and the highest in the ‘Industrialized’ pattern (8427 (sd 3753) kJ/d (2014 (sd 897) kcal/d); *P*<0·05; Table 3). Significant differences were also seen across DP for fat intake (*P*<0·05); the ‘Industrialized’ pattern had the highest intake (82·4 (sd 44·4) g/d) and the ‘Traditional’ pattern had the lowest (55·2 (sd 41·6) g/d). The highest fibre intake was seen in the ‘Traditional’ pattern (26·4 (sd 22·0) g/d) and the lowest in the ‘Industrialized’ (18·2 (sd 13·0) g/d) and ‘Modern’ patterns (17·7 (sd 13·2) g/d; *P*<0·05). No significant differences were found across patterns in the intake of total carbohydrate or protein (g/d). The highest contribution to total energy intake from carbohydrate was observed in the ‘Traditional’ DP (58·2 (sd 12·3) %), whereas the lowest contributions to total energy intake from protein and fat were observed in the ‘Industrialized’ (12·4 (sd 4·0) %) and ‘Traditional’ (28·1 (sd 11·0) %) patterns, respectively (*P*<0·05).

**Table 3:** Daily intakes of energy, macronutrients and fibre, by dietary pattern, among school-aged children (5–11 years) from the 2012 Mexican National Health and Nutrition Survey (*n* 2751)

	Traditional^a^ (*n* 752)		Industrialized^b^ (*n* 541)		Varied^c^ (*n* 1154)		Modern^d^ (*n* 304)	
	Mean	sd	Mean	sd	Mean	sd	Mean	sd
Total energy (kJ)	7037^b,c^	3707	8427^a,d^	3753	7912^a^	3573	7247^b^	2837
Total energy (kcal)	1682^b,c^	886	2014^a,d^	897	1891^a^	854	1732^b^	678
Carbohydrates								
Grams	249·5	141·6	265·3	130·1	249·6	116·9	232·4	92·7
% of total energy	58·2^b,c,d^	12·3	51·9^a^	9·2	52·3^a^	10·9	53·5^a^	8·7
Protein								
Grams	58·0	35·2	60·8	27·3	63·9	40·3	60·9	27·6
% of total energy	13·6^b^	4·6	12·4^a,c,d^	4·0	13·4^b^	4·8	14·1^b^	3·4
Fat								
Grams	55·2^b,c,d^	41·6	82·4^a,d^	44·4	74·5^a,d^	43·5	64·9^a,b,c^	33·7
% of total energy	28·1^b,c,d^	11·0	35·8^a,d^	8·0	34·2^a^	9·0	32·4^a,b^	7·4
Fibre								
Grams	26·4^b,c,d^	22·0	18·2^a^	13·0	18·5^a^	11·8	17·7^a^	13·2

^a,b,c,d^Mean values were significantly different between the dietary patterns, adjusted for the survey design (*P*<0·05, adjusted by Bonferroni method).

As for fruit and vegetables, the ‘Varied’ DP contained most of these food groups compared with the other patterns (consumption of both food groups was 125·6 g/d).

The relative likelihood of having a ‘Varied’ and ‘Modern’ rather than a ‘Traditional’ DP among schoolchildren living in the North region was 38 % (relative risk (RR)=0·62; 95 % CI 0·4, 0·9) and 50 % (RR=0·50; 95 % CI 0·3, 0·8), respectively, compared with the corresponding relative likelihood among schoolchildren living in the South region (Table 4). As for area of residence, the relative likelihood of having an ‘Industrialized’, ‘Varied’ and ‘Modern’ rather than a ‘Traditional’ DP among children living in urban areas was 2·3 (95 % CI 1·6, 3·3), 1·6 (95 % CI 1·2, 2·1) and 2·5 (95 % CI 1·6, 3·8), respectively, compared with the corresponding likelihood among schoolchildren living in rural areas.

**Table 4:** Associations between dietary patterns and sociodemographic and economic factors in school-aged children (5–11 years) from the 2012 Mexican National Health and Nutrition Survey (*n* 2751)

	Industrialized		Varied		Modern	
	RR	95 % CI	RR	95 % CI	RR	95 % CI
Region						
South	1·0	Ref.	1·0	Ref.	1·0	Ref.
Centre	1·2	0·8, 1·8	1·1	0·8, 1·6	1·2	0·8, 1·8
North	0·9	0·6, 1·5	0·6*	0·4, 0·9	0·5*	0·3, 0·8
Area						
Rural	1·0	Ref.	1·0	Ref.	1·0	Ref.
Urban	2·3*	1·6, 3·3	1·6*	1·2, 2·1	2·5*	1·6, 3·8
Indigenous population						
Yes	1·0	Ref.	1·0	Ref.	1·0	Ref.
No	2·7*	1·2, 6·0	2·9*	1·8, 4·7	22·0*	5·1, 93·6
SES						
Low	1·0	Ref.	1·0	Ref.	1·0	Ref.
Middle	3·4*	2·3, 5·1	2·6*	1·8, 3·7	2·2*	1·3, 3·7
High	6·2*	3·9, 9·9	4·5*	3·0, 6·6	3·9*	2·1, 7·3

RR, relative risk; SES, socio-economic status; Ref., reference category. When every variable was analysed, the model was adjusted by the rest of the sociodemographic variables.

* Significantly different from the reference, adjusted for the survey design (*P*<0·05).

The relative likelihood of having a ‘Varied’ and ‘Modern’, rather than a ‘Traditional’ DP, among schoolchildren who did not belong to an indigenous group was three times (RR=2·9; 95 % CI 1·8, 4·7) and twenty-two times higher (RR=22·0; 95 % CI 5·1, 93·6), respectively, compared with the corresponding relative likelihood among schoolchildren who belonged to an indigenous group. The relative likelihood of having an ‘Industrialized’, a ‘Varied’ and a ‘Modern’ *v*. a ‘Traditional’ DP among schoolchildren from the highest SES tertile was 6·2 (95 % CI 3·9, 9·9), 4·5 (95 % CI 3·0, 6·6) and 3·9 (95 % CI 2·1, 7·3), respectively, compared with the corresponding likelihood among schoolchildren from the lowest SES tertile.

## Discussion

In the present cross-sectional analysis based on ENSANUT-2012, four DP were identified by cluster analysis in Mexican schoolchildren aged 5–11 years: ‘Traditional’ (Mexican diet), ‘Industrialized’, ‘Varied’ and ‘Modern’. The prevalence of these patterns varied by sociodemographic variables. All patterns were associated with SES. Notably, the ‘Industrialized’ pattern tended to be consumed more by children in middle and high SES, while the ‘Modern’ pattern was most associated with urban areas; and the strongest association was observed for this ‘Modern’ DP and non-indigenous population. This shows that DP in Mexico are influenced by sociodemographic factors, as was found in another study^(^ ^24^ ^)^.

It is important to note that we did not identify a ‘healthy’ pattern seen in many other studies^(^ ^25^ ^–^ ^27^ ^)^. Although a ‘Varied’ DP was identified, it did not meet the characteristics of a ‘healthy diet’ (adequate, balanced, varied and with the required nutrients for health)^(^ ^28^ ^)^. Even though the ‘Varied’ DP did contain more fruit and vegetables than other DP, it did not reach the daily recommendation for fruit and vegetables proposed by the WHO (at least 400 g/d)^(^ ^29^ ^)^. Furthermore, this pattern also included less healthy foods, such as high-fat mixed dishes made from tortilla or corn.

On the other hand, the ‘Traditional’ pattern in the current study, which was most prevalent in rural areas, had fibre and fat intakes closer to the recommendations of the Institute of Medicine (adequate fibre intake for children aged 5–8 years: 25 g/d; for 9–11-year-old males: 31 g/d; for 9–11-year-old females: 23 g/d)^(^ ^30^ ^)^ and the FAO (fat intake of 25–35 % relative to total energy intake)^(^ ^31^ ^)^ than the other patterns.

Previous studies using principal components analysis have identified patterns among schoolchildren in other countries^(^ ^26^ ^,^ ^27^ ^,^ ^32^ ^,^ ^33^ ^)^. Some of these patterns include: a healthy diet (high in fruit, vegetables and fish, low in saturated fats and refined sugars); a diet high in snacks and other unhealthy energy-dense foods; and other traditional patterns (reflecting the characteristics of the culture where the study was conducted)^(^ ^27^ ^)^.

As expected, the highest intakes of dietary fibre, and lowest intakes of energy and fat, were seen in the ‘Traditional’ DP, while the highest energy and fat intakes were observed in the ‘Industrialized’ pattern. Our findings are consistent with those identified in an earlier analysis of Mexican schoolchildren in ENSANUT-2006, including a ‘rural’ pattern (corn tortilla, legumes, cereals (bread, whole-wheat bread and rice), and low intake of sweets). This pattern had the highest fibre intake (18·2 g/d) and the lowest total fat intake (28·9 g/d) compared with the other patterns reported^(^ ^34^ ^)^.

Further, results from ENSANUT-2006 showed that the Mexico City region was associated with high intakes of energy and fat, while carbohydrate intake was higher in the North^(^ ^35^ ^)^. Although both surveys were designed to represent the Mexican population, the study sample, dietary assessment methodology and organization of the regions differed. Therefore, it is necessary to interpret any comparisons across these surveys with caution.

With the recent nutrition transition^(^ ^36^ ^)^, the traditional Mexican diet has been modernized, with increasing consumption of refined foods, foods of animal origin, saturated fat and sugar, and with decreasing amounts of fibre^(^ ^1^ ^,^ ^37^ ^)^. This is reflected in a segment of Mexican schoolchildren reporting intake of a ‘Modern’ pattern, characterized by foods that are easy to prepare such as tortas, sandwiches and sugary breakfast cereals; as well as in the ‘Varied’ pattern with the consumption of sweetened breads and in the ‘Industrialized’ pattern with the consumption of fast food, desserts, pastries and sweets, as well as snacks made from flour.

Our analysis showed that schoolchildren from urban areas were more likely to consume an ‘Industrialized’ DP, which, given its higher sugar and energy content, is likely to increase the risk of overweight and obesity.

On the other hand, the ‘Modern’ DP may also increase the risk of overweight and obesity, due to low-quality, less nutrient-dense foods. For example, a previous prospective study of DP in 5–9-year-old European children showed a direct relationship between diets with energy-dense nutrient-poor foods and increased fatness in childhood^(^ ^25^ ^)^. Similarly, another study of Mexican children found that DP characterized by sugar-rich foods and sweetened beverages significantly increased the risk of overweight or obesity^(^ ^34^ ^)^.

The change towards less healthy patterns in Mexico may be explained by changes in the food supply, as well as dynamics of the urban area, where the lifestyle is faster and individuals prefer foods that are convenient and do not require preparation^(^ ^9^ ^,^ ^38^ ^)^. Furthermore, it is important to note that the school environment plays an important role in food choices^(^ ^39^ ^)^. A study showed that 43 % of 6–13-year-old Mexican children consumed foods at school, followed by 13 % on the street. Compared with overall daily energy consumed away from home, a disproportionate amount of energy from salty snacks was consumed away from home^(^ ^40^ ^)^. The availability of energy-dense, nutrient-poor foods at school and its surrounding areas contributes to energy intake and diet quality^(^ ^41^ ^)^. In Mexico, the percentages of energy intake from sugar-sweetened beverages and from products high in saturated fat and/or added sugar are higher at school than at home^(^ ^42^ ^)^. Food products oriented to and easily available to children tend to be less healthy, contributing to risk of childhood obesity^(^ ^43^ ^)^.

Given sufficient food availability and intake, the traditional indigenous Mexican diet has been described as a good source of energy, carbohydrates, protein (beans and rice), vitamins, minerals and fibre^(^ ^44^ ^)^. In contrast, newer ‘Western’ diets (highly processed foods, with refined cereal grains, excessive sugar, excessive saturated and *trans*-fats)^(^ ^45^ ^)^ are associated with obesity and other health conditions. We found that school-aged children of households with high SES were less likely to report a ‘Traditional’ DP than those with lower SES.

Socio-economic disadvantage has been widely associated with lower consumption of fruits and vegetables, and higher consumption of energy-dense foods: studies in Europe found a positive association between SES and healthy DP^(^ ^46^ ^)^. A systematic review of dietary intake in adults from low- and middle-income countries found that those with high SES were more likely to consume healthy foods such as lean meats, whole grains, fish and low-fat dairy products, while those with low SES tended to consume more fat and less fibre^(^ ^47^ ^)^.

The differences in results between Mexico and these countries may be due to differences in the trajectory of the nutrition transition and in overall accessibility to healthy foods. Also, it could be due the differences in criteria that are used in other studies to define SES (in some countries, SES could be defined based on educational level, income and/or employment status). In Mexico, higher fibre and lower fat intakes are still seen among those with lower *v*. higher SES^(^ ^17^ ^)^. It is well known that families with lower SES have less money to spend on food, while higher SES families may use certain foods as a status symbol^(^ ^11^ ^)^. The trend towards lower-quality foods as status items is of critical importance, as these foods have lower dietary fibre and other naturally occurring nutrients than a varied traditional diet.

There are some limitations that must be considered in interpreting our results. The patterns are based on a single 24 h recall per child, which limits characterizing the usual diet of individuals, although this should not affect the group-level estimates of the DP. Under-reporting is inherent in studies assessing diet. In the present study, the mother (or person in charge of feeding the schoolchild) reported the diet for her/his child. That person may not be fully aware of foods consumed at school. It is also possible that when the child or his/her caregiver is obese, the caregiver may under-report actual consumption, as has been noted in the literature^(^ ^48^ ^,^ ^49^ ^)^, but this has not been well characterized in Mexico. Another limitation is that the 24 h recall used in the present study has not been validated in the Mexican population and there is an inherent probability of underestimation of energy intake with this kind of methodology; however, this method proved valid to estimate energy intake in children in other studies^(^ ^50^ ^,^ ^51^ ^)^.

Despite limitations, the present study has several strengths. First, the sample was designed to be representative of the Mexican population, so the results are generalizable to the country. Another strength is that although the 24 h recall was not validated in this population, it was carefully administered by trained interviewers, using multiple steps to maximize accuracy^(^ ^52^ ^)^. The information was captured directly, preventing subsequent capture errors, thereby decreasing information bias.

Use of different methods to determine DP may make comparisons with other studies difficult. However, principal components analysis and cluster analysis, the most frequently used methods^(^ ^53^ ^)^, have been shown to generate similar patterns^(^ ^54^ ^)^. Cluster analysis was a strength of our study, as it allowed us to quantitatively describe the actual mean food group intakes of distinct groups. In contrast, PCA generates a score for each factor derived^(^ ^53^ ^)^, based on the intercorrelation of variables rather than the separation of individuals into groups. It can be less interpretable, as actual diets are not described.

On the other hand, although there is evidence that an *a priori* approach to DP (in which dietary indices are constructed based on prevailing dietary recommendations) can show stronger association with health results^(^ ^55^ ^)^, to our knowledge there is not a validated *a priori* approach for Mexican children with details of the recommendation for every food group. Hence, we consider that an *a posteriori* approach can be a good option to analyse the dietary information in the population of the present study.

## Conclusion

In the current analysis of dietary data of Mexican schoolchildren who participated in the ENSANUT-2012, we found that DP were influenced by sociodemographic variables. The ‘Industrialized’ DP was associated with high SES and the ‘Modern’ DP with urban area and non-indigenous status. These DP (‘Industrialized’ and ‘Modern’) can potentially have a negative influence on nutritional intake and are likely contributing to the recent rapid increases in overweight and obesity among children.

Based on our results, parents should be encouraged to retain healthy traditional foods although the family may move towards higher SES and an urban lifestyle, and decision makers should design interventions that allow improvement in the quality of the diet of schoolchildren to prevent overweight and obesity.

## Supplementary material

For supplementary material accompanying this paper visit https://doi.org/10.1017/S1368980017003299. click here to view supplementary material
