# Supplementary material for: Sociodemographic factors are associated with dietary patterns in Mexican schoolchildren
Source: Public Health Nutr. 2017 Dec 1;21(4):702–10. doi: 10.1017/S1368980017003299 (PMC5851048; doi:10.1017/S1368980017003299)
Supplement: Supplementary file 1 [file S1368980017003299sup001.doc]

| Supplemental Table 1. Food group classification | | | | |
| --- | --- | --- | --- | --- |
|
|  | **Food groups*** |  | **Foods** |  |
|  | **Tortilla** |  | Corn tortilla |  |
|  |  |  | Flour tortilla |  |
|  | **Legumes** |  | Any legume |  |
|  | **Egg** |  | Any species |  |
|  | **Sugar-sweetened beverages** |  | Juice drinks made from any natural and prepared fruit |  |
|  |  |  | Sports and energy drinks |  |
|  |  |  | Atole with water, coffee or tea with sugar |  |
|  |  |  | Aguas frescas^‡^ |  |
|  |  |  | Yakult |  |
|  | **Bread and other cereals (excludes corn, rice, pasta and cereals with sugar)** |  | Oatmeal  Whole grain cereal  Salty Bread and non-sweet whole grain cereals |  |
|  | **Milk drinks with sugar** |  | Atole any flavor, smoothies any flavor, flavored milk, |  |
|  |  |  | Milk beverages with sugar, industrialized smoothies |  |
|  | **Snacks made from flour, corn or potato** |  | Chips, popcorn, potato sticks, etc. |  |
|  | **Fast food** |  | Burrito, gringa, hamburger, hot dog, pizza, etc. |  |
|  | **Desserts, pastries and sweets** |  | Desserts |  |
|  |  |  | Candies  Ice cream |  |
|  |  |  | Pastries and cakes (pancakes) |  |
|  | **Industrialized Beverages** |  | Industrialized, soft drinks any flavor, juice drinks |  |
|  | **Meals made of tortilla or corns dough** |  | Mainly mexican food as tacos, sopes, quesadillas, etc. |  |
|  | **Cereals with sugar** |  | Mexican sweet breads, sugary cookies any kind. |  |
|  | **Meat and sausage** |  | Meat of any kind |  |
|  |  |  | Organ meats/meat other organs |  |
|  |  |  | Sausages |  |
|  | **Dairy drinks** |  | Modified milk fat content and/or sugar, whole and/or soy, lactose-free, goat milk, formula |  |
|  | **Fruits** |  | Any kind of fruit |  |
|  | **Rice and pasta** |  | Rice (any preparation) |  |
|  |  |  | Pasta (any preparation) |  |
|  | **Tortas and sandwich** |  | Includes ham croissant, mollete^†^, sandwich any kind°, tortas any kind°, etc. |  |
|  | **Breakfast cereal with sugar** |  | Any Brand: Chococrispis, Zucaritas, Nesquik, Zucoso, Froot Loopss etc. |  |
|  | **Vegetables based stews** |  | Stew made of any vegetables |  |
|  | **Vegetables** |  | Any kind of vegetables |  |
|  | **Fish and seafoods** |  | Any preparation |  |
|  | **Yogurt** |  | Danonino, solid yogurt of any kind |  |
|  | **Drinkable yogurt** |  | Dairy drink with sugar (not solid) |  |
|  | **Cheeses** |  | Cheeses of any kind, any fat |  |
|  | **Juices** |  | Natural fruit juices and vegetables |  |
|  | **Soups and broths** |  | Soups and broths of any kind, including industrialized |  |
|  |  |  | Cream soups based on milk |  |
|  | **Potato** |  | Potato, other tuber, any preparation |  |
|  | **Seeds and oils** |  | Peanut, nut, seed of any kind, oils, margarine, avocado. |  |
|  | **Miscellaneous** |  | Sauce and seasonings |  |
|  |  |  | Dressings and creams |  |
|  |  |  | Emulsifiers |  |
|  | **Drinking water** |  | Drinking water |  |
|  | **Drinks, unsweetened** |  | Coffee or tea without sugar |  |
|  | **Diet sodas** |  | Diet sodas any flavor |  |
|  |  |  | Flavored water (light) |  |
|  |  |  | Powdered beverage |  |
|  |  |  | Mineral water |  |
|  | **Supplements and dietary supplement** |  | Any supplements and dietary supplement |  |
| *Foods were grouped according to the mayor nutrients or common usage. | | | |  |
| ^‡^ Traditional Mexican beverages usually prepared with fruit, water and sugar. | | | |  |
| ^†^ A Mexican dish made of bread that could be salty (with beans and cheese) or sweet (jam or spreadable butter) | | | |  |
| ° It can be prepared of egg, meat, chicken, beans, sausages, etc. | | | |  |
